# A Genetically Confirmed Case of ATR-X Syndrome Without Alpha-Thalassemia: First Case Reported From Jordan

**DOI:** 10.7759/cureus.88943

**Published:** 2025-07-28

**Authors:** Suliman Aljaafreh, Ayman Alhwayan, Atwa Altawarh, Moath Altarawneh, Ruba Alhazaimeh, Abeer Abdalnabi, Eman Alquraan, Sleman Alabdallat, Sumaia Alrababah, Maher Khader

**Affiliations:** 1 Pediatric Department, Royal Medical Services, Queen Rania Children's Hospital, Amman, JOR

**Keywords:** alpha-thalassemia, atr-x syndrome, chromatin remodeling disorders, intellectual disability, whole-exome sequencing

## Abstract

Alpha-thalassemia X-linked intellectual disability syndrome (ATR-X syndrome) is a rare genetic disorder caused by mutations in the *ATRX* gene, typically affecting males and presenting with neurodevelopmental and systemic manifestations. We report, to the best of our knowledge, the first genetically confirmed case of ATR-X syndrome in Jordan, involving a two-and-a-half-year-old male patient who presented with global developmental delay, dysmorphic facies, hypotonia, and bilateral cystic kidneys. Despite persistent microcytic anemia, hemoglobin electrophoresis and PCR for alpha-globin gene deletions were negative. Whole-exome sequencing (WES) identified a hemizygous pathogenic variant in the *ATRX* gene: c.7365C>T (p.Arg2465Cys), confirming the diagnosis. This case contributes to the global understanding of ATR-X syndrome by emphasizing that alpha-thalassemia may be absent in certain mutations. Notably, the presence of progressive renal abnormalities broadens the known phenotypic spectrum. Our findings underscore the importance of genomic diagnostics, especially in resource-limited settings, for accurate identification and early intervention in rare syndromic presentations.

## Introduction

Alpha-thalassemia X-linked intellectual disability syndrome (ATR-X syndrome) is an uncommon neurodevelopmental condition that primarily affects males. Patients present with intellectual disability, distinct facial features, and varying degrees of hematologic abnormalities. This syndrome is linked to pathogenic variants of the *ATRX* gene located on Xq13.3, which encodes for chromatin remodeling proteins responsible for heterochromatin formation, gene regulation, and maintaining genomic integrity. The typical underlying mutations (missense, nonsense, and fragment replacements) lead to loss of function, thus explaining the diverse clinical features seen in patients [1,2].

In ATR-X syndrome, the presence of alpha-thalassemia is often due to epigenetic silencing of the alpha-globin genes. ATRX and other proteins target specific elongation motifs, governing DNA methylation and histone modification. Loss of ATRX function is related to reduced alpha-globin transcription and mild microcytic anemia. Alpha-thalassemia is best diagnosed with HbH inclusion staining, especially when molecular testing for primary alpha-thalassemia is negative [3].

Affected males are usually diagnosed during infancy, as they show signs of hypotonia and developmental delays, followed by intellectual disability and severe difficulties in verbal communication. Other distinct features include microcephaly, hypertelorism, a flat nasal bridge, and coarse facies, which facilitate clinical recognition [4]. Additional findings include seizures, gastrointestinal dysmotility [5], urogenital anomalies such as micropenis or undescended testes [6], and brain abnormalities such as cerebral atrophy or corpus callosum thinning [7].

Clinical suspicion must be followed by molecular confirmation to confirm the diagnosis. Detection is remarkably improved by whole-exome sequencing (WES) when the presentation is atypical or in the absence of alpha-thalassemia [8]. Specific domain mutations correspond with the severity of the phenotype, i.e., some variants in the plant homeodomain (PHD)-like domain exhibit more severe neurologic and urogenital manifestations [6].

The spectrum of presentation is broad. Consistent findings are developmental delays and dysmorphic features, but their severity, along with the combination of other findings, varies significantly between individuals. Notably, the absence of alpha-thalassemia increases the chance of delayed diagnosis, which is problematic in regions with limited genomic testing [9].

Such limited awareness, alongside the constrained access to genomic testing in some areas [10], suggests that ATR-X syndrome is underdiagnosed. With fewer than 200 reported cases worldwide [11] and an estimated prevalence below one per million [9], other differential diagnoses include syndromes like Fragile X, Coffin-Lowry, or *MECP2*-related disorders that also involve X-linked intellectual disabilities [12].

A multidisciplinary approach, incorporating developmental therapy, nutritional support, and hormone replacement therapy when necessary, provides effective supportive management. Although individuals with significant comorbidity may experience a more variable prognosis, most reach adulthood with appropriate care [13].

To the best of our knowledge, this is the first case documented as genetically confirmed ATR-X syndrome in Jordan for a two-and-a-half-year-old male patient known to have classic phenotypic features, persistent microcytic anemia, and developmental delays. Anemia is apparent. However, molecular testing for alpha-thalassemia was negative, which accentuates the importance of genomic analyses in cases with atypical presentation. There is a need to document the diagnostic spectrum in underrepresented populations, as this case emphasizes and contributes to the knowledge of the rare syndrome and its diverse clinical features worldwide.

## Case presentation

A two-and-a-half-year-old male patient with profound global developmental delay, along with persistent hypotonia and recurrent episodes of respiratory and feeding difficulties, was brought to the clinic. He is the first child of a non-consanguineous Mediterranean couple, without any concerning family history of neurological or genetic disorders.

On clinical exam, he appeared malnourished, along with developmental advancement below what is generally expected in a child of this age. Growth parameters include height 87 cm (4th percentile, z-score: -1.75), weight 9.5 kg (<1st percentile, z-score: -2.4), body mass index (BMI) 12.5 (z-score: -2.1), and head circumference 46 cm (2nd percentile, z-score: -2.0), consistent with global growth restriction. Vital signs were stable, with a heart rate of 118 beats per minute, blood pressure of 100/65 mmHg, respiratory rate of 25 breaths per minute, temperature of 36.7°C, and oxygen saturation of 97% on room air.

Systemic physical examination showed an overall hypoactive child with limited movements and responsiveness. He had characteristic craniofacial dysmorphism, including hypertelorism (increased distance between the eyes), epicanthic folds, a broad and flat nasal bridge with anteverted nares, tenting of the upper lip with a thin border, and a reduced chin (retrognathia), along with low-set ears. He had a mild coarsening facial appearance, which is characteristic of ATR-X syndrome. Additional craniofacial findings were a high-arched palate and areas of skin with a marbled appearance (cutis marmorata) on the limbs and trunk. On chest examination, air entry was regular and symmetrical, with no added sounds or signs of respiratory distress. Cardiac auscultation revealed no abnormal murmurs or gallops. Examination of the abdomen revealed a soft, non-distended, and non-tender state, with no palpable masses or hepatosplenomegaly. The genital examination noted bilateral undescended testes with hypospadias.

The patient demonstrated significant global developmental delay, was nonverbal, and did not display purposeful motor actions or social engagement, traits classic to ATR-X syndrome. Neurologically, the patient presented striking truncal hypotonia with inadequate head support and retracted, effortless movement of the upper and lower limbs.

At birth, the patient was admitted to the neonatal intensive care unit (NICU) for 10 days due to hypotonia and poor oral intake. He was delivered prematurely at 35 weeks via cesarean section, with a birth weight of 2.4 kg. On examination, he appeared mildly tachypneic and had a wide anterior fontanelle, along with bilateral undescended testes. During his NICU stay, a renal ultrasound revealed bilateral kidney hypoplasia, measuring 2.7 cm on the right and 2.5 cm on the left, with multiple cysts, the largest measuring 4 × 4 mm. A brain MRI showed nonspecific bilateral white matter hyperintensities, thinning of the corpus callosum, and mild ventricular dilation. Cardiac evaluation and thyroid function tests, including TSH (thyroid-stimulating hormone) and free T4 (thyroxine), were within normal limits.

The child was readmitted at one month of age due to feeding difficulties and decreased activity. A full sepsis workup was performed, and he was discharged after receiving supportive care. At the age of two months, he was noted to have persistent microcytic anemia, along with cutis marmorata and retrognathia. On follow-up, hypotonia and a high-arched palate were also documented. A repeat renal ultrasound showed enlargement of a previously identified cyst on the left kidney, now measuring 8 × 8 mm, with no evidence of hydronephrosis or structural obstruction.

Concerns about developmental status around five months of age rise when the child fails to engage in eye contact or visually track objects. The ophthalmological assessment was conducted, and sluggish pupillary responses were observed with standard optic discs and maculae. There was a marked delay in all areas of development, which included no vocalization, poor motor skills, and severe hypotonia. By nine months, physiotherapy and orthotics were implemented to address neuromuscular deficits. He remained nonverbal and nonambulatory as of his most recent appointment.

The child had several hospitalizations due to respiratory tract infections at three, 16, and 22 months. During one of these admissions, a neurology consult noted that the child had persistent hypotonia but no signs of neurodegenerative regression. Between the ages of one and two years, progressive renal imaging changes were observed, including increased echogenicity and the development of bilateral cysts. A dimercaptosuccinic acid (DMSA) scan showed scarring of the left kidney with decreased function and preserved function of the right. Investigations into metabolism, including plasma amino acids and urine organic acids, as well as serum ammonia, were routine. Evaluation of peripheral blood showed microcytic anemia (Hb 10.5 g/dL, MCV 65 fL) but with a normal hemoglobin electrophoresis profile (HbA2 2.9%, HbF 1.1%). These findings, particularly the persistent microcytic anemia despite negative alpha-globin PCR, in conjunction with the renal abnormalities, suggested a syndromic condition and prompted WES. Table 1 below summarizes the laboratory and imaging investigations.

**Table 1:** Summary of Laboratory and Imaging Investigations Renal imaging reveals structural abnormalities, including bilateral cysts and reduced left kidney function, supporting renal involvement. Brain MRI findings (white matter hyperintensities, corpus callosum thinning) are consistent with neurodevelopmental abnormalities seen in ATR-X syndrome. Persistent microcytic anemia with normal alpha-globin PCR supports the diagnosis without structural alpha-thalassemia. Hb: hemoglobin; MCV: mean corpuscular volume; TSH: thyroid-stimulating hormone; FT4: free thyroxine; DMSA: dimercaptosuccinic acid; PCR: polymerase chain reaction; MRI: magnetic resonance imaging; CBC: complete blood count; HbF: hemoglobin F; HbA2: hemoglobin A₂.

Investigation	Parameter	Result	Reference Value
Renal Ultrasound	Right kidney size	4.7 cm	6.2 ± 0.8 cm
	Left kidney size	4.2 cm	6.2 ± 0.8 cm
	Bilateral cysts	Present	Absent
	Largest cyst (left kidney)	1 × 0.8 cm	None
	Echogenicity	Increased	Normal
	Hydronephrosis	Absent	Absent
DMSA Scan	Left kidney scarring	Present	Absent
	Left kidney function	32%	45–55% per kidney
	Right kidney function	68%	45–55% per kidney
Brain MRI	White matter signal	Hyperintensities	Normal
	Corpus callosum	Thinned, 1–15 mm	3–5 mm thickness
	Ventricles	Enlarged	Normal size
CBC	Hemoglobin	10.5 g/dL	11.5–13.5 g/dL
	MCV	65 fL	75–87 fL
Hb Electrophoresis	HbA2	2.9%	1.5–3.5%
	HbF	1.1%	<2%
Alpha-globin PCR	Structural deletions	Negative	No deletions
Thyroid Function	TSH	3.6 mIU/L	0.7–5.7 mIU/L
	FT4	1.2 ng/dL	0.7–1.7 ng/dL
Metabolic Screen	Plasma amino acids	Normal	Age-specific patterns
	Urine organic acids	Normal (no abnormal peaks)	No abnormal peaks
	Ammonia	40 μmol/L	15–45 μmol/L

Genetic analysis identified a hemizygous pathogenic variant in the *ATRX* gene, specifically NM_000489.6:c.7365C>T (p.Arg2465Cys), which is apparently present in exon 9. Pathogenic ATR-X syndrome has been documented to harbor this missense variant. The genetic findings, in conjunction with the clinical picture, revealed no features of structural alpha-thalassemia, confirming the diagnosis of ATR-X syndrome without alpha-globin gene deletion. The systemic findings are presented in Table 2.

**Table 2:** Summary of Systemic Findings This table summarizes multisystem involvement, consistent with ATR-X syndrome. Neurologic and craniofacial abnormalities, combined with renal and genitourinary findings, align with the known phenotypic spectrum of the condition. The genetic variant confirms diagnosis in the absence of alpha-globin gene deletions. MRI: magnetic resonance imaging; PCR: polymerase chain reaction; *ATRX*: alpha-thalassemia/mental retardation syndrome X-linked gene.

System	Findings
Neurologic	Global developmental delay, truncal hypotonia, no seizures, periventricular white matter hyperintensities, corpus callosum thinning on MRI
Hematologic	Persistent microcytic anemia, negative alpha-globin PCR
Renal	Bilateral renal cysts, increased echogenicity, left kidney scarring, and reduced function
Genitourinary	Hypospadias, bilateral undescended testes
Craniofacial	Retrognathia, flat nasal bridge, high-arched palate, cutis marmorata
Ophthalmologic	Sluggish pupillary response, normal optic disc and macula
Genetics	Hemizygous pathogenic *ATRX* variant: c.7365C>T (p.Arg2465Cys)

The patient is actively participating in multidisciplinary treatment with neurology, nephrology, endocrinology, physiotherapy, occupational therapy, and speech-language therapy. Cryptorchidism, which requires surgical removal, is also under hormonal assessment. He is not experiencing any clinical comorbidities and remains under coordinated follow-up. Genetic counseling has been delivered to the family, highlighting the uniqueness and heterogeneous presentation of the ATR-X syndrome.

## Discussion

ATR-X syndrome is an uncommon X-linked disorder first documented by Gibbons et al. [1]. It is characterized by intellectual disability, distinctive craniofacial features, urogenital anomalies, hypotonia, and often microcytic anemia due to alpha-globin gene silencing [3].

Our patient has displayed the hallmark phenotypic features of ATR-X syndrome, which include global developmental delay, characteristic facial dysmorphism [4], urogenital dysgenesis, and microcytic anemia. Most strikingly, the microstructural alpha-globin gene mutation, included within the scope of clinically diagnosed anemia, was found to be harmful, accounting for 10-15% of ATR-X cases where microcytic anemia exists without structural alpha-thalassemia [2]. Unlike the classical presentation of alpha-thalassemia with HbH inclusions, which occurs in 75-80% of cases [3].

The progressive nature of the cystic changes (from 4 x 4 mm at birth to 1 x 0.8 cm at 2.5 years) expands the known phenotypic spectrum of ATR-X syndrome. The renal manifestations of the patient with bilateral cysts and increased echogenicity, along with scarring of the left kidney with reduced function of 32%, are notable. Renal abnormalities are observed in only 10-15% of cases of ATR-X syndrome [14], and cystic kidneys are present in approximately 5-8% of cases [7]. Other clinical features in this patient are aligned with other described ATR-X cases: growth restriction was seen (height 4th percentile and weight below 1%) in 70-85% of cases, the distinct craniofacial traits in more than 90% of cases, severe developmental delay in 95%, hypotonia in 85-90%, and genital deformities in roughly 80% [12]. Neuroimaging "white matter hyperintensities, thin corpus callosum, and enlarged ventricles also correspond with the literature reports" [7], while the absence of seizures is notable despite significant CNS structural abnormalities.

The mutation p.Arg2465Cys (c.7365C>T) we identified in one of our patients lies within the ATRX-DNMT3-DNMT3L (ADD) domain of chromatin remodeler *ATRX* that is responsible for binding to nucleosomes. The most striking feature resulting from the lack of this domain in patients with this mutation is that severe intellectual disability occurs in 90-95% of cases, absent or severely limited speech development in 85-90% of the cases, and severe motor delay in 80-90% of the cases [14], which fully supports the clinical scenario of our patient. When considering comparisons between other *ATRX* mutations, p.Arg2465Cys shows some distinct patterns: increased incidence of microcytic anemia without detectable HbH inclusions (40% vs. 10-15% overall), slightly greater renal abnormality rate (15-20% vs. 10-15% overall), and a lower proportion of patients with seizures (25-30% vs. 30-35% overall) [12]. This mutation is likely to alter chromatin remodeling activity in a way that is less detrimental to alpha-globin gene regulation than mutations in other domains, which explains the absence of structural alpha-thalassemia despite the presence of microcytic anemia [14]. Compared to mutations in the PHD-like domain [6], our patient's mutation presents with typical neurological severity but distinct hematological and renal manifestations.

Highlighting the importance of genomic technology in identifying rare neurodevelopmental disorders, the diagnosis was made via WES [8]. The ATR-X syndrome is reported in the literature, highlighting the lack of acknowledgment of the syndrome in resource-constrained scenarios due to the absence of genomic technologies [10].

Supportive and multidisciplinary approaches remain the mainstay for the management of the disorder, requiring integrated input from different disciplines to deal with hypotonia, feeding issues, renal problems, and developmental delays [13]. Despite the generally observed significant intellectual disability, developmental outcomes have been noted to improve after the commencement of early therapeutic intervention.

This case illustrates the occurrence of ATR-X syndrome without structural alpha-thalassemia and significant renal anomalies, thereby contributing to the existing literature on the syndrome. As the first report with documented renal anomalies, this case strengthens the phenotypical variability of the syndrome. This also warrants considering a diagnosis of ATR-X syndrome in children presenting with intellectual disability, syndromic features, and no typical hematological changes, thus emphasizing the need for broadened diagnostic criteria. The understanding of certain mutations in the *ATRX* gene and their specific clinical expressions, which assist in predicting the course and treatment of this pathology, aids in widening the understanding of the condition through cross-examination of the genotype-phenotype relationship.

## Conclusions

This case illustrates the phenotypic variability of ATR-X syndrome, which includes the lack of structural alpha-thalassemia and the presence of renal anomalies. To the best of our knowledge, it marks the first genetically confirmed case from Jordan, demonstrating the potential of WES for diagnosing rare neurodevelopmental disorders in challenging environments. Accurate and early genetic diagnosis facilitates early multidisciplinary intervention, as well as optimized clinical management and family counseling, thus improving outcomes and quality of life. These findings also emphasize the greater need to integrate genomic testing into clinical practice, enabling the identification of individuals with ATR-X syndrome and similar disorders.
